# Establishment and Characterization of a Newly Established Diabetic *Gerbil* Line

**DOI:** 10.1371/journal.pone.0159420

**Published:** 2016-07-18

**Authors:** Xiaohong Li, Jing Lu, Ying Wang, Xueyun Huo, Zhenkun Li, Shuangyue Zhang, Changlong Li, Meng Guo, Xiaoyan Du, Zhenwen Chen

**Affiliations:** School of Basic Medical Science, Capital Medical University, Beijing Key Laboratory of Cancer Invasion & Metastasis Research, Beijing 100069, China; Max-Delbrück Center for Molecular Medicine (MDC), GERMANY

## Abstract

**Objectives:**

We aimed to selectively breed a spontaneous diabetic *gerbil* when a sub-line of inbred *gerbil* showed increased blood glucose levels was found recently. Then we investigated the characteristics including the serum insulin, triglyceride, cholesterol, *leptin*, *adiponectin* and explored the underlying molecular mechanism for the diabetic phenotype.

**Methods:**

The spontaneous diabetic line of *gerbil*s was selectively inbreed the sub-line of *gerbil* by monitoring blood glucose of each animal. The serum insulin, *adiponectin*, and *leptin* levels were tested using an ELISA kit. The expression levels of *GLUT4*, *Akt*, *leptin*, *adiponectin*, and *calpain 10* (*CAPN10*) were tested by western blot and Quantitative Real-time PCR (qPCR) in liver, skeletal muscle, and white adipose.

**Results:**

Our results show that the percentages of animals with FPG≥5.2 (mmol/l), PG2h≥6.8 (mmol/l) and both FPG≥5.2 and PG2h≥6.8 (mmol/l) were increased with the number of breeding generations from F0 (21.33%) to F6 (38.46%). These diabetic *gerbils* exhibited insulin resistance and *leptin* resistance as well as decreased *adiponectin* level in the serum. We also observed decreased expression of *adiponectin* and increased expression of *leptin* in the skeletal muscle, respectively.

**Conclusions:**

These results indicate that we have primarily established a spontaneous diabetic *gerbil* line, and the diabetic phenotypes may have been accounted for by altered expression of *leptin* and *adiponectin*.

## Introduction

Diabetes is a global public health issue and the number of people with diabetes is expected to increase by 42% (from 51 to 72 million) in industrialized countries between 1995 and 2025 and by 170% (from 84 to 228 million) in industrializing countries [1]. Diabetes also represents a major public health concern in China and the overall prevalence of diabetes was estimated to be 11.6% in Chinese adults [2]. Type 2 diabetes mellitus (T2DM) accounts for more than 90% of cases of diabetes [3]. The hallmark of T2DM is the development of insulin resistance [4], whereas type 1 diabetes is defined as insulin deficiency.

Animal models of T2DM are urgently needed in order to better understand the pathogenesis and potential therapeutic targets. The existing animal models of diabetes include those experimentally induced, spontaneous, and genetically modified mice, rats and minipigs [5, 6]. Experimental induced models are often established by streptozotocin treatment or high-fat diet feeding, which are time consuming. Furthermore, genetically modified models could only mimic limited features of T2DM in human. For example, *db/db* mice and *ob/ob* mice can only represent phenotypes of losing the single gene of *leptin* or its receptor [7]. Spontaneous model is very useful and valuable especially in studying genetic factors of diabetes; but such model is not readily available.

As a potential diabetic model, the *Mongolian gerbils* have been reported by Boquist L et al who found that some *gerbils* in their colony had relatively higher fasting blood glucose and obesity [8]. Unfortunately, the percentage of *gerbils* with high blood glucose is very low (3/42, 7.14%). During our study of inbreeding *Mongolian gerbils*, we found that a sub-line of inbred *gerbils* with a incidence of higher blood glucose (21.33%) much higher than what Boquist L et al reported. We went on to selectively inbreed this sub-line to establish a new *gerbil* model of diabetes. In present study, we reported the characterization of the diabetic phenotype in our diabetic *gerbils*.

T2DM has many etiological factors including environmental (diet and lifestyle) and genetic factors. There is an important connection between multiple genes and T2DM [9, 10]. Until now, many genes have been identified to associate with T2DM in genome-wide association studies (GWAS), such as transcription factor 7-like 2 (*TCF7L2*) gene, glucose transporter member 4 (*GLUT4*), and *adiponectin* [11, 12]. T2DM manifests itself with insulin resistance and defect in glucose utilization. In order to characterize the diabetic phenotypes in our spontaneous diabetic *gerbils*, we chose five candidate genes including *GLUT4* and protein kinase B (*Akt*) which participate in glucose uptake[13]; *leptin* and *adiponectin* which are associated with insulin sensitivity [14, 15]; and *calpain 10* (*CAPN10*) which was identified having associations with T2DM[16].

## Methods

### Ethics Statement

All experiments and animal procedures were strictly conducted in accordance with the Guidelines of Capital Medical University Animal Experiments and the Experimental Animals Management Committee. This study was approved by the Animal Experiments and Experimental Animal Welfare Committee of Capital Medical University (Permit Number: 2012-X-38).

### Animals and Housing

The *Mongolian gerbils* were bred and maintained at the animal facility of the Capital Medical University under standard laboratory conditions (room temperature, 20–24°C; relative humidity, 50–70%; 12 h light/12 h dark cycle) with free access to food and water.

### Selective Inbreeding Diabetic *Gerbils*

The experimental animals were placed in individual cages with one pair or one litter. When the animals were 3-month-old(S1 Fig), the fasting glucose (FPG) level as measured and 2h glucose tolerance test (PG2h) was performed using a blood glucose meter (SANNUO, China). All tests were repeated a week after. Then the male and female littermates were mated according to the criterion that both of them with the fasting glucose (FPG)≥5.2 (mmol/l)and 2h glucose tolerance (PG2h)≥6.8(mmol/l), as considering inbreeding depression, we set this standard FPG≥5.2 which was a little lower than Boquist L et al reported(8). When the breeding yielded offsprings, we tested the FPG and PG2h of the offsprings and continued to mate with the inbreeding method. When the animals were to 1–1.2 years old and had offsprings (S1 Fig), we euthanized the *gerbils* and tested a series of physiological and biochemical indexes, including insulin, *leptin* and *adiponectin* level in the serum. Tissues were harvested to pathological analysis. We repeated this process from F0 generation to F6 generation. The daily food intake of diabetic *gerbils* (n = 15)and control *gerbils* (n = 15) were tested for 8 days by weighing food every day for individual cage of every animal and calculated the average value reduction of food.

### Selection of Animals and Tissue Preparation

When the animals were 1–1.2 years old and had offsprings, before being euthanized, the animals were tested for the FPG after 16h fasting; then the *gerbils* were anesthetized, whole blood samples were collected into anticoagulant tubes and non-anticoagulant tube from the orbital sinus. The serum in non-anticoagulant tube were separated and frozen at -80°C for ELISA tests. Plasma separated from anticoagulant tubes and serum were using for biochemical analsyis by Synchron cx5 (Beckman, USA) and MEK-7222K (NIHON KOHDEN, Japan). After blood collection, the *gerbils* were killed by giving an overdose of pentobarbital. Skeletal muscle, adipose tissue, liver, kidney and pancreas were collected. Each collected tissue was divided into three portions, one of which was fixed in formalin for histological analysis; two were stored at -80°C for Quantitative Real-time PCR (qPCR) and Western blot analysis.

### Insulin, Glucose Tolerance Test and the Measurement of Insulin, *Leptin* and *Adiponectin* levels in Serum

Insulin tolerance test was performed by insulin (Novolin, China) intraperitoneal injection (0.75 U/kg) after 4h fasting. Glucose tolerance test was performed after 16 h fasting and *gerbils* were given glucose orally (2 g/kg). Then blood samples were collected from the tail tip at 0, 30, 60, and 120 min after glucose administration and were measured for blood glucose levels using a blood glucose meter (SANNUO, China). The serum levels of insulin, *adiponectin* and *leptin* were measured using 10 μl and 50 μl serum according to the instructions of the ELISA kits (Millipore, Germany abcam, USA abcam, USA). We used a microplate reader (BioTek, USA) reading the absorbance at a wavelength of 450 nm and calculate the value using the generated logistic curve-fit.

### Histological Analysis

Skeletal muscle, adipose tissue, liver, kidney and pancreas were fixed on 4% paraformaldehyde for about 2 week. Then the five tissues were processed using routine histology procedures, paraffin embedding, and 2 μm-thick slices were cut and placed on glass slides. The paraffin sections were stained with hematoxylin and eosin (HE) and then examined microscopically. The individual(s) performing the histological examination were blinded for the animal information.

### Rapid-amplification of cDNA Ends (RACE)

To obtain the full-length cDNA of 5 genes (*GLUT4*, *Akt*, *adiponectin*, *leptin* and *CAPN10*), total RNA was obtained from an outbred *gerbil*. The PCR primers (Table 1) were designed within the conservative fragments of the *gerbil* gene based the homology comparison. We used the full-length cDNA for the gene clone. Genes were cloned using a 5′ RACE System for Rapid Amplification of cDNA Ends kit (Invitrogen, USA) and a SMARTer^™^ RACE cDNA Amplification Kit (Clontech, USA) to receive gene’s 3′ as the protocols described. Fragment assembly was accomplished by using the DNAMAN 5.5 software (DNAMAN, USA). And DNA sequencing (Zhuandaoshengwu, China) and sequence analysis were performed by using the DNA Star v 7.1 software (DNA Star, USA).

**Table 1:** The primer sequences to amplified the conservative fragment in cloning the sequence of 5 candidate genes including *GLUT4*, *Akt*, *Leptin*, *Adiponectin*, and *CAPN10* and the resulted lengths of conservative fragment.

Name of Gene	GenBank accession number	Primer	Sequence	Product length (bp)	Total length (bp)
*GLUT4*	KT377191	Forward Reverse	CTCAGTGGTTGGGAAGGAAAAGG GCCCTAAGTATTCAAGTTCTG	994	1486
*Akt*	KT377189	Forward Reverse	ATGARCGACGTRGCMATYGTGAA CCTCAGGCYGTSCCRCTGGC	1344	2363
*Leptin*	KT377190	Forward Reverse	CACCAAAACCCTCATCAAGAC AGAGHGARGCTTCCAGGAC	329	634
*Adiponectin*	KT377188	Forward Reverse	CTTCTCTCCAGGAGTGCCATCTCTG TACTGGTCGTAGGTGAAGAGAAC	344	1169
*CAPN10*	KT377192	Forward Reverse	TTCCCSGCYTCRGASTCCTCGCT TTGSAGGGAAAGCARCTGTTGTT	1038	2467

### Quantitative Real-time PCR (qPCR)

Total RNA from muscle, adipose tissues, and liver from diabetic and control *gerbil* was extracted using the Trizol reagent (Tiangen, China), and cDNA was generated from 2 μg RNA in a 20μl reaction mixture according to the manufacturer’s protocols (Tiangen, China). Specific primers to each target gene were designed using Primer-BLAST (S1 Table). Real-time was performed using the CFX Real-Time PCR system (Bio-Rad, USA) in accordance with following protocols: pre-denaturation at 95°C for 15 min, 40 cycles of incubation at 95°C for 10 s, annealing and extension at 60°C for 35 s, and 71 cycles of melt curve analysis at 60°C for 10 s. Real-time quantitative amplification analysis was carried out by Bio-Rad CFX software (Bio-Rad, USA).

### Western Blotting

The proteins were extracted from samples using the Proteins Extraction Kit (CWBIO, China) as following step: added in 500μl cold tissue protein extraction reagent buffer (CWBio, China) to tissues and the tissue debris was removed by centrifugation at l0 000 rpm and 4°C for 15 min. Then the total protein concentrations were determined using a BCA kit (CWBio, China). The proteins (30 μg) were separated by 12% or 15% sodium dodecyl sulfate polyacrylamide gel electrophoresis at 160V. The separated proteins were transferred to 0.22 μm Nitrocellulose membranes (PALL, USA) at 0.2A for 2 h and incubated for 1 h at room temperature with 5% skim milk in Tris-buffered saline and Tween 20. The primary antibodies used include *leptin* (R&D, 1:1000 dilution), *adiponectin* (abcam, 1:1000 dilution), *GLUT4* (Cell Signaling Technology, 1:1000), *Akt* (Cell Signaling Technology, 1:1000), *CAPN10* (abcam, 1:1000 dilution) and *GAPDH* or beta-actin (Cell Signaling Technology, 1:1000). All antibodies were diluted in 0.5% skim milk solution. After overnight incubation with the primary antibody in 4°C, the membranes were washed and incubated with the horseradish peroxidase-conjugated goat anti-rabbit or anti-mouse IgG antibodies (Baltimore Pike, 1:5000) for 1 h at room temperature. The membranes were washed 3 times using Tris-buffered saline and Tween 20 once for 10 minutes. The signals were detected by ECL and quantified using Image Lab software (Bio-Rad, USA).

### Statistical Analysis

All data were analyzed by student’s *t*-test. The differences were considered significant when the *p* value was less than 0.05. All results were expressed as means± S.D. of all independent experiments. Statistical analysis was carried out using the SPSS 16.0 (SPSS Inc., USA).

## Results

### Establishment of diabetic inbred *gerbil* line

**Fig 1:**
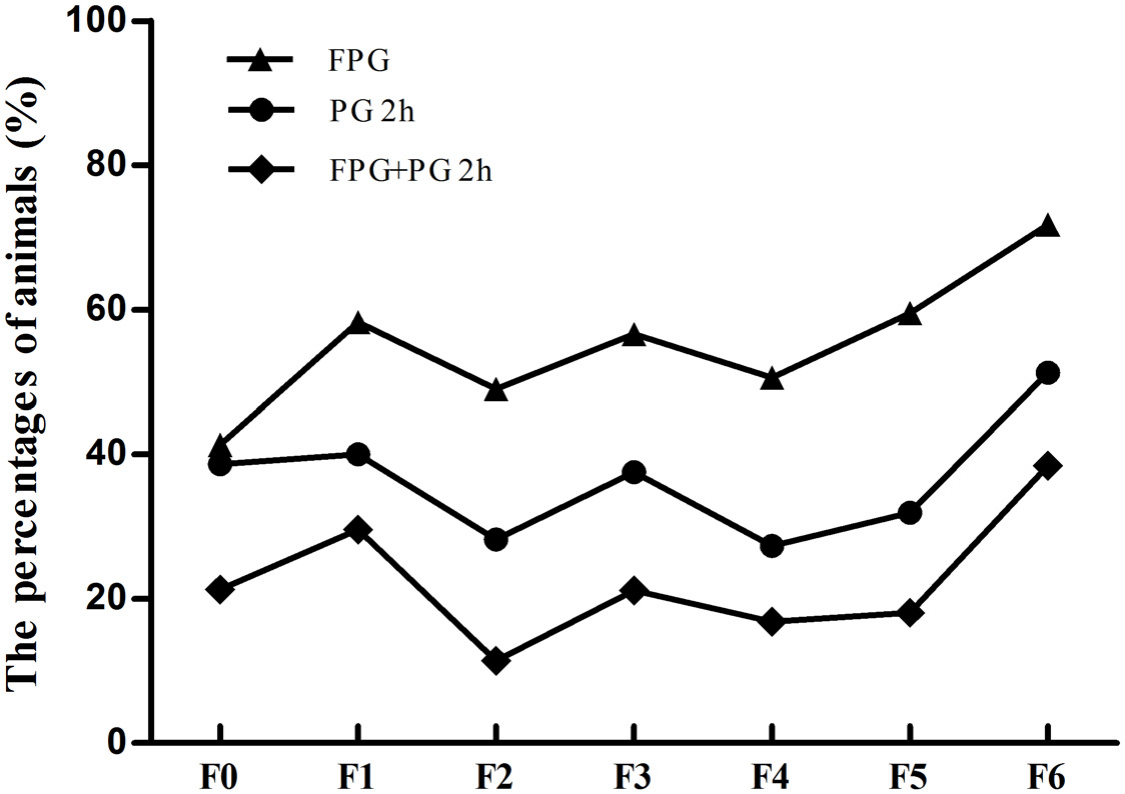
The percentages of animals with FPG≥5.2, PG2h≥6.8 and both FPG≥5.2and PG2h≥6.8 (mmol/l). Animals of FPG≥5.2, PG2h≥6.8 and both FPG≥5.2and PG2h≥6.8 were increased with the number of breeding generations from F0 to F6.

After finding that the incidence of high blood glucose was relatively higher in a sub-line of inbreeding *gerbils*, we selectively bred the group by choosing animals with FPG≥5.2 and PG2h≥6.8 (mmol/l) when they were 3 months old and mating them with inbreeding method. We tested a total of 855 *gerbils* including 75, 115, 149, 189, 172, 116, and 39 *gerbils* from the F0, F1, F2, F3, F4, F5, and F6 generations, respectively. The blood glucose analysis results of FPG≥5.2 and PG2h≥6.8 (mmol/l) percentages from F_0_ to F_6_ are show in Table 2. We found that the percentage of animal with FPG≥5.2 (mmol/l) in F0, F1, F2, F3, F4, F5, and F6 were 41.33%, 58.26%, 48.99%, 56.61%, 50.58%, 59.48%, and 71.79%, respectively. The percentage of animal with PG2h≥6.8 (mmol/l) in F0, F1, F2, F3, F4, F5, and F6 were 38.67%, 40.00%, 28.19%, 37.57%, 27.33%, 31.90%, and 51.28%, respectively. The percentage of animals with both FPG≥5.2 and PG2h≥6.8 (mmol/l) in F0, F1, F2, F3, F4, F5, and F6 were 21.33%, 29.57%, 11.41%, 21.16%, 16.86%, 18.10%, and 38.46%, respectively. Our results showed that the incidence of animals with FPG≥5.2 (mmol/l), PG2h≥6.8 (mmol/l) and both FPG≥5.2 and PG2h≥6.8 (mmol/l) animals were increased with the number of breeding generations from F0 to F6 (Fig 1). The percentages of animals with FPG≥5.2 (mmol/l) was increased more quickly compared the other two indexes. Although the increase of percentage with both FPG≥5.2 and PG2h≥6.8 (mmol/l) were relatively slow (Fig 1), the trend was increasing and the percentage in F6 was 38.46%, which was far more than that in F0 (21.33%). Furthermore, more than 50% animals in F6 were complied with the standard (FPG≥5.2). These results indicated that the spontaneous diabetic *gerbil* line has been primarily established.

**Table 2:** The number of animals tested blood glucose, with FPG≥5.2, PG2h≥6.8 (mmol/l), and both FPG≥5.2 and PG2h≥6.8 (mmol/l). The numbers in parentheses are the percentages of each number in each generation.

generation	total animal number	FPG>5.2	PG2h>6.8	FPG>5.2 and PG2h>6.8
D-F_0_	75	31(41.33%)	29(38.67%)	16(21.33%)
D-F_1_	115	67(58.26%)	46(40.00%)	34(29.57%)
D-F_2_	149	73(48.99%)	42(28.19%)	17(11.41%)
D-F_3_	189	107(56.61%)	71(37.57%)	40(21.16%)
D-F_4_	172	87(50.58%)	47(27.33%)	29(16.86%)
D-F_5_	116	69(59.48%)	37(31.90%)	21(18.10%)
D-F_6_	39	28(71.79%)	20(51.28%)	15(38.46%)

### The changes in the serum levels of triglyceride, cholesterol, insulin, *leptin* and *adiponectin*

**Fig 2:**
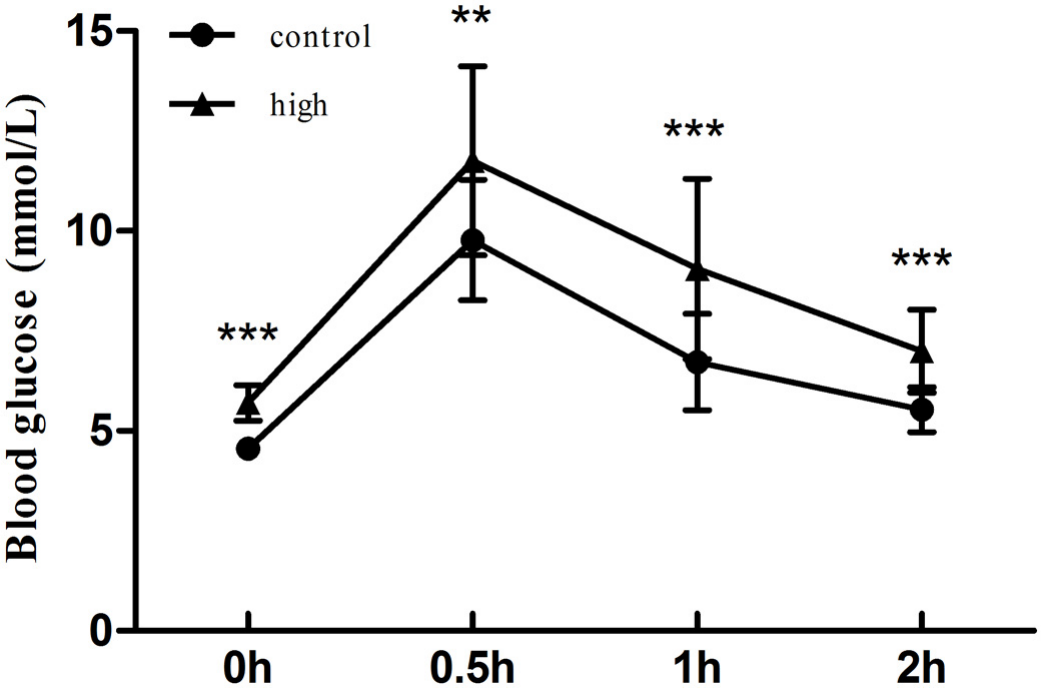
The glucose tolerance test between control and experiment group. The glucose tolerance tested at their 3 months old showed glucose intolerance in the experimental group. Notes: “*” (*p*≤0.05), “**” (*p*≤ 0.01), and “***” (*p*≤0.001) showed significantly different between experimental and control animals.

We randomly chose 20 *gerbils* (male n = 9, female n = 11) with high blood glucose as the experimental group, whose FPG were more than 5.2 mmol/l and PG2h were more than 6.8 mmol/l; and 20 general inbreeding *gerbils* (male n = 10, female n = 10) as control group. And the glucose tolerance of all these gerbils tested at their 3 months old showed glucose intolerance in the experimental group (Fig 2). The serum insulin, *leptin* and *adiponectin* levels were measured using ELISA kits and the serum triglyceride, cholesterol and fasting glucose levels were measured by using Synchron *cx5*. The results showed that fasting glucose, insulin, homoeostasis model assessment for insulin resistance (HOMA-IR), triglyceride, cholesterol and *leptin* levels in the experimental group were all significantly higher than those in the control group (Table 3). In contrast, the serum *adiponectin* level was significantly lower in experimental group (10.65 ug/ml) than that in control group (23.75 ug/ml) (Table 3). These data demonstrated that the diabetic inbred *gerbils* we selectively bred exhibited insulin and *leptin* resistance. The spontaneous diabetic *gerbil* group we established is T2DM model.

**Table 3:** The FPG, insulin, triglyceride, cholesterol, *leptin*, *adiponectin* and HOMA-IR levels in serum from high blood glucose *gerbil* and control *gerbil*.

Groups	FPG (mmol/l)	Insulin (μU/ml)	HOMA-IR^1^	Triglyceride (mmol/l)	Cholesterol (mmol/l)	*Leptin* (pg/ml)	*Adiponectin* (μg/ml)
control	4.54±0.94	13.61±8.03	4.37±2.98	0.52±0.33	2.52±0.33	288.29±146.25	23.75±4.64
high	9.02±2.40***	232.58±95.09***	70.17±31.32***	4.47±4.06***	5.87±2.44***	22080.41±7346.33***	10.65±3.93***

Notes: Values are means±S.E. from 20 control *gerbils* and 20 high blood glucose *gerbils*.

“*” (*p*≤0.05), “**” (*p*≤ 0.01), and “***” (*p*≤0.001) showed significantly different between experimental and control animals.

“^**1**^” HOMA-IR = fasting insulin (μU/ml)×fasting glucose(mmol/l)/22.5

### The insulin tolerance test between control and experimental group

**Fig 3:**
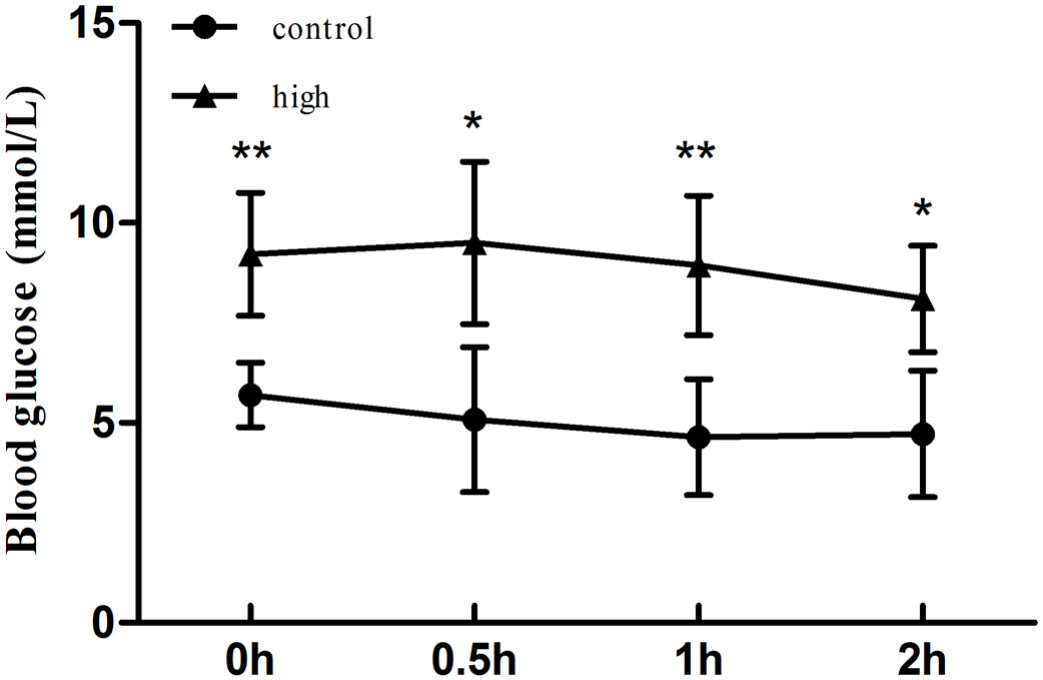
The insulin tolerance test between control and experiment group. The glucose tolerance tested at their 1–1.2 years old showed decreased tolerance to insulin in the experimental group. Notes: “*” (*p*≤0.05), “**” (*p*≤ 0.01), and “***” (*p*≤0.001) showed significantly different between experimental and control animals.

In order to further confirm insulin resistance in T2DM Mongolian *gerbil*, not type 1 diabetes mellitus (T1DM), we randomly chose 10 *gerbils* (male n = 5, female n = 5) with high blood glucose as the experimental group and 10 general inbreeding *gerbils* (male n = 5, female n = 5) as control group aged 1–1.2 years old. The result showed that compared with control group, experimental group exhibited decreased tolerance to insulin (Fig 3). It further confirmed that the spontaneous diabetic *gerbil* group we established is T2DM model.

### Pathological analysis of the diabetic target organs

**Fig 4:**
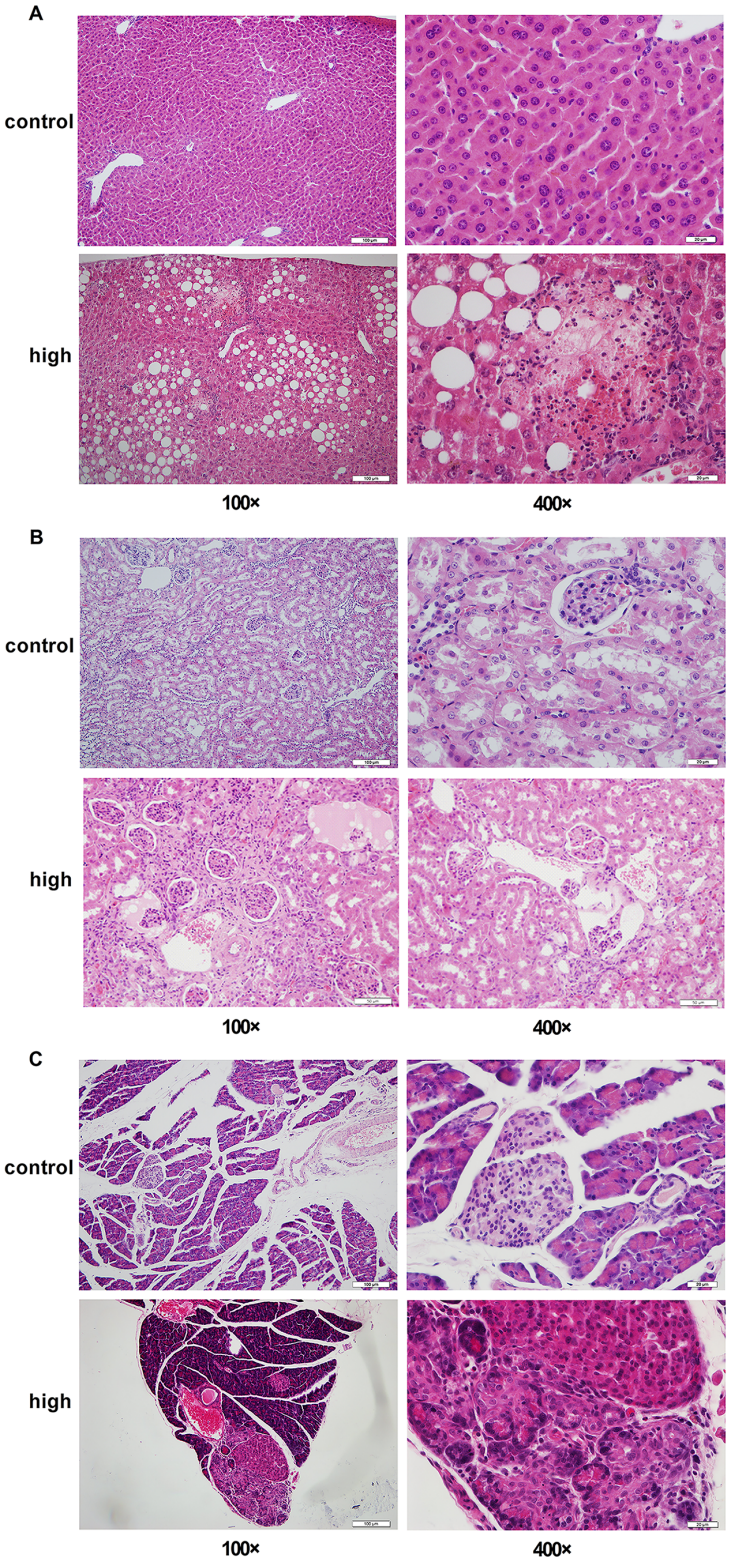
The pathological analysis of target organs between control group and experimental group using HE stain (100×, 400×): liver (A), kidney (B), and pancreas (C). (A): in liver, we can find severe hepatic steatosis and focal necrosis in diabetic *gerbils* was the liver pathological changes.; (B): the kidney are glomerulus atrophy and the tubular can see protein substance, also some of the nucleus pycnosis, cataclastic, and solution in the end in diabetic *gerbils*; (C): the pancreas represent nuclear pyrosis and cell necrosis leading to local necrosis in diabetes *gerbil*. All of these three organs had histological changes between control and experimental group.

To investigate the target organs of this spontaneous diabetes model, we examined the histological changes of skeletal muscle, adipose tissue, liver, kidney and pancreas by HE stain in diabetic *gerbils* and the control *gerbils*. Our results showed that compared with the control *gerbils*, the diabetic *gerbils* had pathological changes in the liver, kidney and pancreas, but not in the skeletal muscle and adipose tissue. In the liver, we found marked hepatic steatosis and focal necrosis in the diabetic *gerbils* (Fig 4A). The injury in the diabetic kidney is glomerulus atrophy and tubular protein accumulation (Fig 4B). The pancreas of the diabetic *gerbils* showed nuclear pyrosis and cell necrosis leading to local necrosis (Fig 4C).

### Molecular cloning and homology analysis of five candidate diabetic genes from *gerbils*

**Fig 5:**
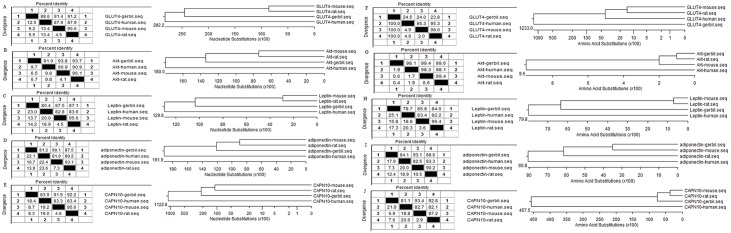
Analysis CDS of the five genes with DNAStar. We analyzed the sequences with the ORFs of *Mus musculus* and *Rattus norvegicus*, along with the human, and lined the sequence distances and drew the phylogenetic tree to analyze their homology and the expression level of the five genes. (A-E) shows the results of the *GLUT4*, *Akt*, *leptin*, *adiponectin*, and *CAPN10* DNA sequence distances analysis and their phylogenetic tree; (F-J) shows the results of the *GLUT4*, *Akt*, *leptin*, *adiponectin*, and *CAPN10* protein sequence distances analysis and their phylogenetic tree.

In order to understand the molecular mechanism for the diabetic phenotypes in *gerbils*, we chose to clone five candidate genes, including *GLUT4*, *Akt*, *leptin*, *adiponectin*, and *CAPN10*, by RACE (Table 1). The full-length sequences of these genes were submitted to GenBank and their accession numbers are shown in Table 1. We analyzed the sequences of the cDNAs of the *gerbils* and compared them with those of mice (*Mus musculus*), rats (*Rattus norvegicus*), and humans (*Homo sapiens*). In order to analyze their homology, we performed sequence distance analysis and drew the phylogenetic tree. We found that the sequences of the cDNAs of the 5 genes in *gerbils* were similar to the other mammalian species, while the *Akt* (91.0%, 90.9%, and 96.1% similarity in nucleic acid sequences with human, mouse, and rat) represented the greatest similarity among the 5 genes. *GLUT4*, *leptin*, *adiponectin*, and *CAPN10* showed similar homology (Fig 5A–5E). Similar results were obtained when the amino acid sequences were analyzed, except that the human *GLUT4* had the lowest similarity with *gerbil* (24.5%) (Fig 5F–5J).

### Analysis of the expression of candidate diabetic genes

**Fig 6:**
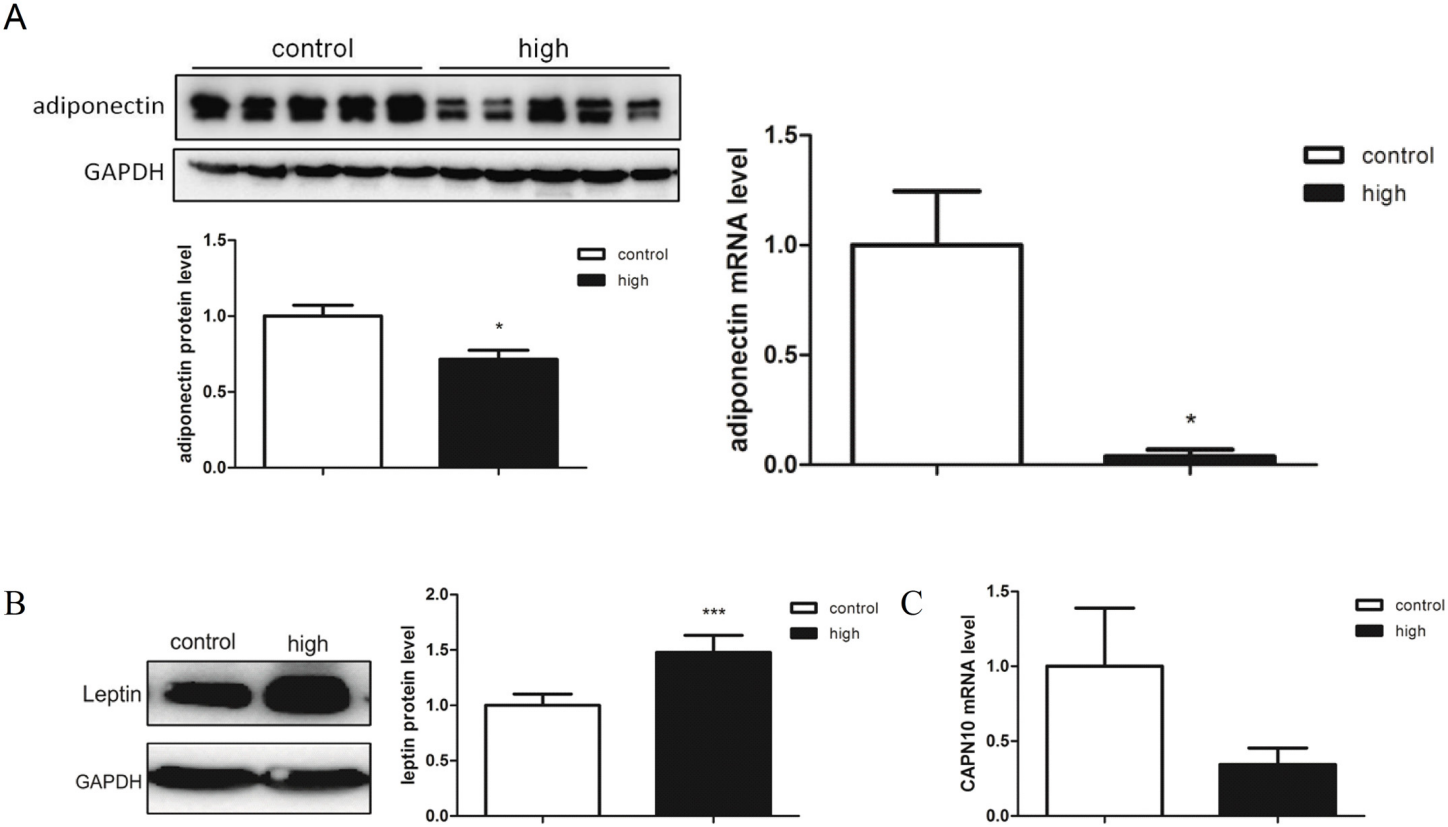
Expression level of the diabetic related genes between control and experimental gerbils with western blot and RT-qPCR. (A) was the *adiponectin* expression changes at protein and mRNA level, both of them were significantly lower in high blood glucose group than that in control group; (B) was the *letpin* expression change at protein level was significantly higher in high blood glucose group than that in control group; (C) was the *CAPN10* expression change at mRNA level, it had a tendency of lower expression in high blood glucose group. Notes: “*” (*p*≤0.05), “**” (*p*≤ 0.01), and “***” (*p*≤0.001) showed significantly different between experimental and control animals.

We performed qPCR and Western blot to measure the mRNA and protein expression of the above mentioned five candidate diabetic genes in the liver, skeletal muscle and white adipose. The results showed that the expression level of *adiponectin* was significant lower in the diabetic *gerbils* than control *gerbils* in the skeletal muscle at both mRNA and protein levels (Fig 6A). *Leptin* expression was significantly higher in the skeletal muscle of diabetic *gerbils* at the protein level (Fig 6B). *CAPN10* showed a tendency of lower expression at the mRNA level in the skeletal muscle (Fig 6C), but the difference did not reach statistical significance. The expression of *Akt* was not affected in any of these tissues. These results suggested regulation of *leptin* and *adiponectin* may have contributed to the diabetic phenotype in our diabetic *gerbils*.

## Discussion

Animal models of T2DM are essential for diabetes research. The existing T2DM models have their limitations. For example, the *C57BL/6* mice are sensitive to high-fat diet induced T2DM, but it takes 3–4 months to induce typical diabetes [17]. Thus, more animal models are urgently needed to further investigate the mechanism of diabetes. As a spontaneous diabetic model, our diabetic *gerbil* could be used directly and time-saving of inducing with chemicals and diet. *Mongolian gerbils* were reported to be a potential model of diabetes. Nakama K. reported that *gerbils* can be established as a diabetic model after feeding on a diet containing tolbutamide. The tolbutamide-treated *gerbils* exhibited typical pathological diabetic changes in the pancreas. The increases in blood glucose, free fatty-acid and insulin levels in tolbutamide-treated *gerbils* were more dramatic than rat model of diabetes [18]. Nishigaki R. et al reported that streptozotocin treatment can also induce diabetes in *gerbils* [19]. However, these chemical-induced diabetic models have their limitations in understand the genetic basis of diabetes. While our diabetic *gerbil* is very useful and valuable especially in studying genetic factors of diabetes, for its exhibiting the features of T2DM—insulin resistance and multigenic disease. *Psamomoys Obesus gerbil* is another nutritionally induced diabetic animal model of which transition from native diet to laboratory rodent chow showed hyperinsulinemic and hyperglycemic with marked insulin resistance, and it’s a good model for studying insulin resistance and insulin signaling pathways in muscles, but its characteristics are not sustained [20–22]. While our inbreeding group is selected from generation to generation, its characteristics of insulin resistance, leptin resistance, low adiponectin level and the expression changes of the genes may sustain longer and be hereditary. Similar with other spontaneous diabetic models, our group also could comprehensively study the mechanism of T2DM as a complex polygenic disease.

In this study, we selectively bred a group of spontaneously hyperglycemic diabetic *gerbils*. After analyzing FPG≥5.2 and PG2h≥6.8 (mmol/l) percentages in every generation from F_0_ to F_6_, we found that the percentage of FPG≥5.2 (mmol/l) and PG 2h≥6.8 (mmol/l) is increasing from generation to generation. The results also indicated that as many as 31.58% animals in the F6 generation are diabetic and this incidence is much higher than what Boquist L et al reported [8].

Insulin resistance is the hallmark of T2DM. So we tested the insulin level and found the insulin levels in the diabetic *gerbils* were higher than the control animals. Our measurements of the serum *leptin* level and calculations of the insulin resistance index, as well as insulin tolerance test also suggested that our diabetic *gerbils* exhibited insulin resistance and *leptin* resistance. *Leptin* resistant is synonymous with obesity, as suggested by the observations made by Toshihiro Miura et al and Martin G. et al [23, 24]. In addition, in a cohort study of women, a large amount of T2DM could be attributed to obesity [25]. The examination of *adiponectin* level showed that it was significantly lower in the diabetic *gerbils* than the control *gerbils*. It was reported that reduced *adiponectin* level was associated with insulin resistance, obesity, and T2DM [26]. Therefore, all these data suggested that our diabetic *gerbils* are representative of T2DM.

The diabetic phenotypes of our diabetic *gerbils* were also supported by our histological results showing that diabetic histological changes were observed in the liver, kidney, and pancreas. T2DM is often accompanied by complications and there are more than 100 existed complications, such as kidney complications, neurological complications, and eye complications [27]. Liver is a metabolic organ and pancreas controls the production and secretion of insulin. The histological damages to the liver, kidney, and pancreas in our diabetic *gerbils* may have been secondary to the chronic diabetes.

To further understand the molecular basis of the diabetic phenotype, we measured the expression of some genes that are known to be associated with metabolic disorders. We chose and tested the expression levels of *GLUT4*, *Akt*, *leptin*, *adiponectin*, and *CAPN10* as diabetic related genes. The results showed that *adiponectin* had reduced expression at both the mRNA and protein levels in the skeletal muscle; while *leptin* expression was increased at the protein level in the skeletal muscle. Liu Y. et al. had reported that muscle is a major target tissue for *adiponectin*, an adipokine that increases glucose uptake in the muscle [28]. It was also reported that *adiponectin* can stimulate glucose uptake and fatty-acid oxidation through the activation of *AMPK* in the skeletal muscle, thus, may lead to the increase of cholesterol in serum [29]. There were many reports suggesting that *leptin* regulates fatty acid oxidation through the *AMPK* pathway in the muscle [30]. Although further studies are needed, it is tempting for us to speculate that the spontaneous diabetic phenotypes may have been accounted for by altered expression of *leptin* and *adiponectin*. In the gene expression analysis, *CAPN10* also exhibited tendency of lower expression at mRNA levels in the skeletal muscle of the diabetic *gerbils*. This result is consistent with previous reports that *CAPN10* participated in glucose metabolism and had a decreased mRNA level in the muscle of T2DM animal models [31, 32]. The changes of expression level of three tested genes in the skeletal muscle also suggested that this tissue might be a key target tissue in this diabetic *gerbil* model. As for we tested daily food intake and found that there was no difference between dysglycemic *gerbils* and control *gerbils* (S2 Table), these genes may contribute more to the pathogenesis of diabetic *gerbil*.

In conclusion, we have established a spontaneous diabetic *gerbil* line and the regulation of *leptin* and *adiponectin* genes may be involved in the molecular mechanism of this T2DM model.

## Supporting Information

S1 Fig Adult *gerbil* (left) and 1.2 months-age *gerbil* (right). (TIF)

S1 Table The accession number, primer sequences, annealing temperatures, and the lengths of the PCR products for cloned 5 genes including, *GLUT4*, *Akt*, *Leptin*, *Adiponectin*, and *CAPN10*. (DOCX)

S2 Table The daily food intake between control and high blood glucose *gerbils*. (DOCX)
