# Supplementary figures and images for: Establishment and Characterization of a Newly Established Diabetic Gerbil Line
Source: PLoS One. 2016 Jul 18;11(7):e0159420. doi: 10.1371/journal.pone.0159420 (PMC4948894; doi:10.1371/journal.pone.0159420)

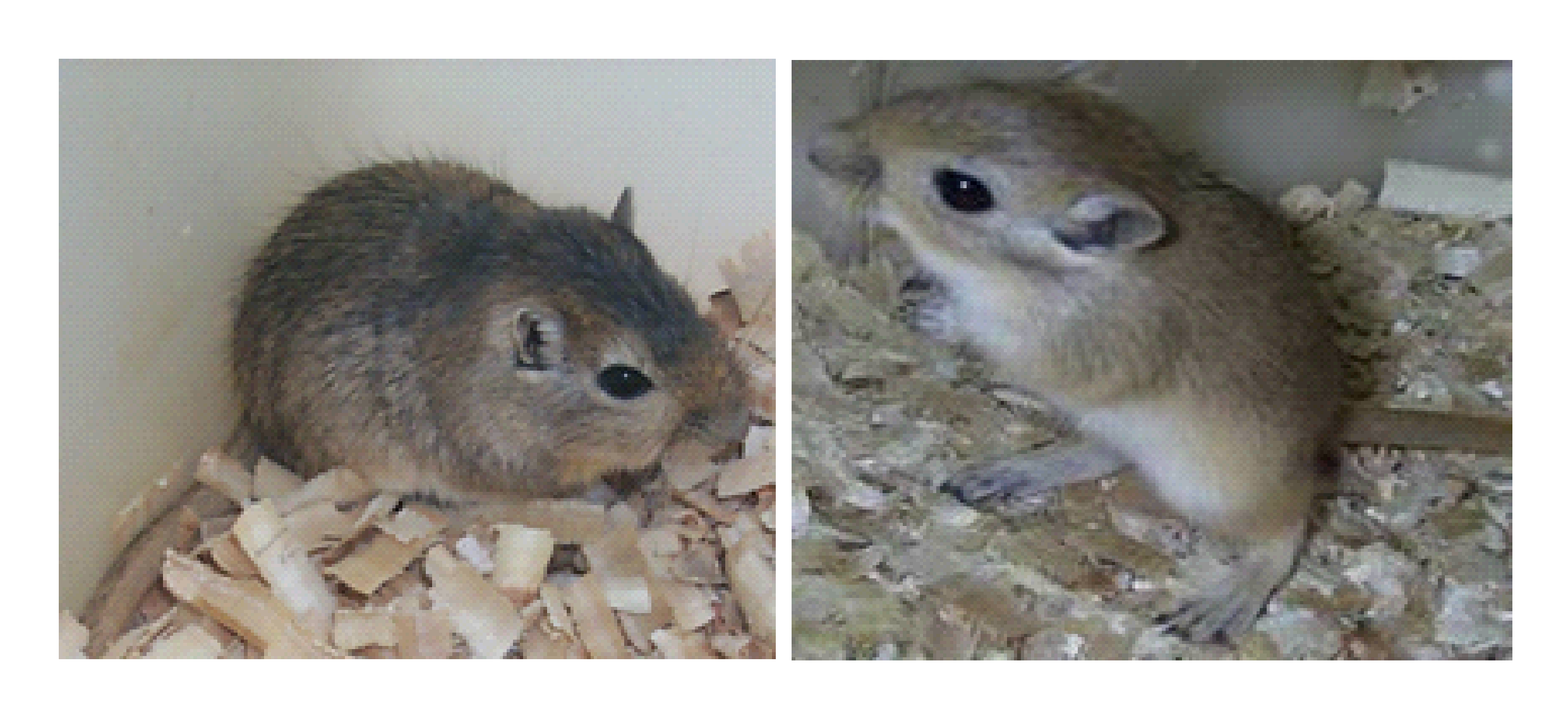

Supplement: S1 Fig — (TIF) [file pone.0159420.s001.tif]
