# Supplementary material for: Establishment and Characterization of a Newly Established Diabetic Gerbil Line
Source: PLoS One. 2016 Jul 18;11(7):e0159420. doi: 10.1371/journal.pone.0159420 (PMC4948894; doi:10.1371/journal.pone.0159420)
Supplement: S1 Table — (DOCX) [file pone.0159420.s002.docx]

**S1 Table. The accession number, primer sequences, annealing temperatures, and the lengths of the PCR products for cloned 5 genes including *GLUT4*, *Akt*, *Leptin*, *Adiponectin*, and *CAPN10*.**

| Name of Gene | Primer | Sequence | Annealing temperature (℃) | Product length  (bp) |
| --- | --- | --- | --- | --- |
| *GLUT4* | Forward  Reverse | CTTCCTTCTATTTGCCGTCC  GGGTTTCACCTCCTGCTCTA | 60 | 142 |
| *Akt* | Forward  Reverse | TGGCTCTCTCCCTCTGGAGT  GACACACAGCAACAAGCCGA | 60 | 114 |
| *Leptin* | Forward  Reverse | TGTGGCTGAAACTGCTGTGCT  CATTGATCCTGGTGACGATGG | 60 | 129 |
| *Adiponectin* | Forward  Reverse | TATCGTTCTGCGTTTAGCGTGG GGCATCATAATGATTCTGTTGGTTG | 60 | 105 |
| *CAPN10* | Forward  Reverse | AGTCCATTCACAGCCAGGAGA  CCAGGCCACAGCAAATACCC | 60 | 106 |
