# Supplementary material for: Establishment and Characterization of a Newly Established Diabetic Gerbil Line
Source: PLoS One. 2016 Jul 18;11(7):e0159420. doi: 10.1371/journal.pone.0159420 (PMC4948894; doi:10.1371/journal.pone.0159420)
Supplement: S2 Table — (DOCX) [file pone.0159420.s003.docx]

| **Groups** | **Food intake**  **(g)** |  |
| --- | --- | --- |
| control | 5.40±1.40 |  |
| high | 4.86±1.80 |  |

**S2 Table. The daily food intake between control and high blood glucose *gerbils***

Notes: Values are means±S.E. from 15 control *gerbils* and 15 high blood glucose *gerbils*.

“*” (*p*≤0.05), “**” (*p*≤ 0.01), and “***” (*p*≤0.001) showed signiﬁcantly different between experimental and control animals.
